# Spirometric Values of Greek People and Comparison with ECSC and GLI Values in COPD People

**DOI:** 10.2174/1874306401812010029

**Published:** 2018-07-31

**Authors:** Nikolaos Tatsis, Sotirios Kakavas, Evgenios Metaxas, Evangelos Balis, George Tatsis, Nikolaos Pantazis, Petros Bakakos, Nikolaos Koulouris, Κostantinos Hadjistavrou

**Affiliations:** 1 Pulmonary Department of EVAGGELISMOS Hospital, Athens, Greece; 2 Department of Hygiene, Epidemiology & Medical Statistics, Athens University Medical School,Athens, Greece; 3 1^st^ Respiratory Medicine Department, National and Kapodistrian University of Athens Medical School, ‘’SOTIRIA” Hospital for Diseases of the Chest, Athens, Greece

**Keywords:** COPD, GOLD classification, GLI equations, ECSC equations, FVC, FEVI

## Abstract

**Background:**

During the past few years, the use of criteria introduced by Global Initiative for Chronic Obstructive Lung Disease (GOLD) is recommended for the diagnosis and classification of Chronic Obstructive Pulmonary Disease(COPD),taking into account the values of a Forced Expiratory Volume In 1 second (FEV1) and a Forced Expiratory Volume In 1 second (FEV1) to Forced Vital Capacity (FVC) ratio. In Europe, the reference values of the European Coal and Steel Community (ECSC), that were originally developed in 1993 are still used.

**Aim of the Study:**

The study aimed to carry out measurement of spirometric values in a healthy, non smoking Greek population, development of local equations and comparison with ECSC and Global Lung Initiative(GLI) equations, in order to see if there is a need for separate ones in everyday use.

**Methods:**

Normal predicted values for FEV1 and FEV1/FVC% were obtained from a group of 500 healthy subjects, aged 18-89 years. In addition, a group of 124 COPD patients, with no other comorbidities was studied. Patients were classified according to GOLD criteria in four groups with ECSC, GLI predicted values or with our own predicted values.

**Results:**

The statistical analysis has revealed that there is no significant difference among the three sets of predicted values and no statistical difference was detected among the classification of COPD patients.

**Conclusion:**

It is shown that the 3 sets of predicted values are almost identical, despite the fact that they have been collected from different study populations.Αccording to the study, there is no need in recalculating values for Greek population.

## 1 INTRODUCTION

Chronic Obstructive Pulmonary Disease (COPD) causes annually 275 million deaths and is the 4^th^ common cause of death worldwide [1].In 2020, it is expected to be the 3^rd^ cause of death [2]. Since 1990, the systematic study and analysis of risk factors, pathogenesis, diagnosis and treatment of patients with COPD are evolving [3].During the past few years, the use of criteria introduced by Global Initiative for Chronic Obstructive Lung Disease (GOLD) is recommended for the diagnosis and classification of COPD [4, 5].

According to these criteria, air flow limitation is defined by a Forced Expiratory Volume In 1 second (FEV1) to Forced Vital Capacity (FVC) ratio less than a pre-specified normal value, while the extent of the decrease of the post-bronchodilation FEV1 defines the severity of the disease. More specifically, the presence of FEV1/FVC < 70% accompanied by symptoms confirms the diagnosis of COPD. These criteria have been introduced and accepted worldwide [6],but at the same time, they are also considered to be arbitrary, at least to some degree [7].Normal reference (or predicted)-values are determined by studying non-smokers who are not suffering from any other comorbidity. The main factors influencing these parameters are race, gender, age and height [8]. Occasionally, weight and BMI have also been used for the calculation of spirometric reference values.

In Europe, the reference values of the European Coal and Steel Community (ECSC) are still used. These values were originally developed in 1983 from a population of North Europe [9] and were finalized in 1993 [10].However, the use of normal or predicted values remains an unsettled issue among scientists, because the universal use of the existing reference values is often criticized, since these values have been collected from a specific population of healthy subjects many years ago. Therefore, it has been argued that there is a constant need for reevaluation of the normal spirometric values [8, 11].In agreement with this notion, this study aimed to carry out measurement of spirometric values in a healthy, nonsmoking Greek population and the subsequent development of local equations for the determination of reference values. In 2008, because of the difficulty in satisfactorily describing the age-related growth and the decline of lung function with age, Stanojevic *et al*. with their work made the first step [12]. Then Quanjer *et al*. in 2012 [13] gave rise to Global Lung Function Initiative, which was endorsed from ERS, ATS, ACCP and many more. Since then, new multiethnic reference values for lung function have been introduced^.^

Furthermore, differences in the classification of subjects, as COPD patients or not, were investigated using either commonly used global equations, GLI and ECSC or equations collected from a group of local healthy subjects, like the ones included in the study.

Truly, race and ethnicity are defined differently in various national contexts [11], and the selection of appropriate reference equations in spirometry and the issue of “race correction” or population-specific norms remain the topics of discussion [14, 15]. In an effort to minimize this phenomenon, we have included only individuals of Greek ethnicity.

## 2 SUBJECTS AND METHODS

The study was designed at the end of 2012 and the spirometric tests were performed from 2013 until the end of 2016. This is the reason why the ECSC values were used as predicted values, since up to then, several numbers of spirometric devices (Including ours) had been using these predicted values. Tests were performed in a sitting position according to (ATS/ERS Taskforce) guidelines [16].Prior to spirometry, calibration check was undertaken daily with a 3-L syringe, and confirming that the variations were within the limits, *e.g.*, ±3% (90 mL). After each test, an immediate on-screen evaluation of major acceptability criteria (including start, duration and end of test) in addition to the automated review was performed by the computer software. As recommended by the ATS/ERS task force, subjects were asked to perform up to a maximum of eight maneuvers in an attempt to obtain reproducible results. The largest forced vital capacity (FVC) and Forced Expiratory Volume In one second (FEV1) were selected.

A group of 500 healthy subjects, with age ranging from 18 to 89 years, were studied. According to the relevant literature [17-25], the sample size of 239 men and 261 women in our group of healthy individuals was more than adequate for the purposes of our study. Healthy individuals were recruited in health screening department of our hospital. The key inclusion criteria [14, 15] were: (I) life-long non-smokers; (II) no symptoms and history of chronic cardiopulmonary diseases (chronic bronchitis, asthma, lung cancer, pulmonary fibrosis, pulmonary tuberculosis, chronic heart diseases, *etc*.); (III) no abnormal findings on physical examination; (IV) written informed consent was obtained. The key exclusion criteria were: (I) upper or lower respiratory infection within 4 weeks; (II) long-term exposure to harmful gas or particles; (III) using β-blocker for treatment; (IV) pregnant women, epileptic; (V) other diseases or surgeries potentially affecting lung function. Health questionnaires about medical history and personal characters were provided to the participants (Table **1a**).

A second group of 124, with age ranging from 24 to 91 years, stable COPD patients, without any other comorbidities, who have visited and monitored by Pulmonary Department in our hospital for almost a duration of 5 years continuously and took the spirometry test at least one time during the study period were enrolled. Diagnosis of COPD and the classification of severity of airflow obstruction were established according to the GOLD guidelines (Table **1b**)

### 2.1 Statistical Analysis

Normal continuous variables were summarized through their mean and Standard Deviation (SD) and comparisons were done using the Student’s t-test. Linear regression analysis was used for the estimation of equations predicting FVC and FEV1. The set of covariates which were examined as potential predictors included age, height, weight and BMI along with their transformations (*e.g.* height squared *etc*.). Normal logarithm changes of FEV1 and FVC were also considered in cases where the best fitting model did not agree with the linear regression assumptions. For instance, the normal logarithm fluctuation/change was considered necessary for the FVC model, since models of the FVC in their original scale presented significant heterogeneity. Moreover, both measurements tended to increase up to the age of 25 years and decrease thereafter.

Thus age was introduced to the models as a piecewise linear term (*i.e*. with different fluctuations before and after the 25^th^ year of age). The concordance between predictions based on the equations from the current study and those based on the ECSC equations was assessed mainly through Lin’s concordance correlation coefficient (ρ_c_). ρ_c_ is used to quantify agreement between two measures of the same variable and ranges from -1 to 1, with 1 indicating perfect agreement.

The predicted values from the current study’s equations were used as reference values for the classification of patients included in the COPD group, according to GOLD criteria [5, 6].In addition, these patients were also classified using the predicted values from ECSC and GLI equations. Agreement regarding COPD staging using different predictive equations was assessed using the weighted Cohen’s Kappa coefficient.

All analyses have been performed using Stata 13.1 (Stata Corp.,TX USA). p-values lower than 0.05 were considered statistically significant.

## 3 RESULTS

From the analysis of measured FEV1, FVC and FEV1/FVC values in healthy individuals, the following predictive equations were obtained:

Study equations for predicted FEV1

Male (R^2^=0.73, Residual SD=0.38)

$\mathrm{FEV1}=\left\{\begin{array}{c} -25.857+0.296\text{∗}H-0.000728\text{∗}H^{2}+0.0285\text{∗}A\;,\mathrm{Age}<25\\ -25.144+0.296\text{∗}H-0.000728\text{∗}H^{2}-0.0288\text{∗}(A-25),\mathrm{Age}\;\geq 25 \end{array}\right.$

Female (R^2^=0.80, Residual SD=0.28)

$\mathrm{FEV1}=\left\{\begin{array}{c} -2.546+0.0343\text{∗}H+0.0082\text{∗}A\;,\mathrm{Age}<25\\ -2.342+0.0343\text{∗}H-0.0232\text{∗}(A-25),\mathrm{Age}\;\geq 25 \end{array}\right.$

FEV1 in liters, age(A) in years, height(H) in cm

Study equations for predicted FVC

Male (R^2^=0.69, Residual SD=0.12)

In (*FVC*)

$\mathrm{FEV1}=\left\{\begin{array}{c} -13.518+0.156\text{∗}H+0.00041\text{∗}H^{2}-0.00138\text{∗}W+0.0196\text{∗}A\;,\mathrm{Age}<25\\ -13.094+0.156\text{∗}H-0.00041\text{∗}H^{2}-0.00138\text{∗}W-0.0070\text{∗}(A-25),\mathrm{Age}\;\geq 25 \end{array}\right.$

Female (R^2^=0.74, Residual SD=0.13)

In (*FVC*)

$=\left\{\begin{array}{c} -11.355+0.130\text{∗}H-0.00035\text{∗}H^{2}-0.00107\text{∗}W+0.0338\text{∗}A\;,\mathrm{Age}<25\\ -10.509+0.130\text{∗}H-0.00035\text{∗}H^{2}-0.00107\text{∗}W-0.0079\text{∗}(A-25),\mathrm{Age}\;\geq 25 \end{array}\right.$

FVC in liters, age (A) in years, height(H) in cm, weight (W) in kgr; ln denotes the natural logarithm function

Study equations for predicted FEV1/FVC

Male (R^2^=0.03, Residual SD=6.82)

*FEV*1/*FVC*86.07-0.0707*A

Female (R^2^=0.08, Residual SD=8.45)

$\mathrm{FEV1}/\mathrm{FVC}\;=\;105.38-0.7852\text{∗}A+0.0069\text{∗}A^{2}$

FEV1/FVC expressed as %, age(A) in years, height(H) in cm

-*FEV1, FVC and FEV1/FVC (%) ratios predictions by ECSC equations were obtained using formulas given in Quanjer 1993^(10)^*

-FEV1, FVC and FEV1/FVC (%) ratios predictions by GLI-2012 equations were obtained using the R “pred_GLI” function from the library “rspiro” (https://github.com/thlytras/rspiro and http://www.ers-education.org/guidelines/global-lung-function-initiative/spirometry-tools/r-macro.aspx

Regression analysis revealed that FEV1 in healthy subjects was on average almost constant between 18-25 years of age and declined linearly thereafter for both men and women. A positive and linear association with height was apparent in women, whereas for men, a quadratic height term was also statistically significant. Age trends were similar for FEV1. However, FVC values had to be transformed to their natural logarithms in order to achieve a valid linear regression model. Quadratic height terms were significant for both men and women. Additionally, weight was negatively associated with FVC (allowing for adjustments in age and height). FEV1/FVC ratios declined linearly for the whole age range in men. In women, the rate of decline in FEV1/FVC ratios was not constant (less steep slopes at higher ages), thus a quadratic age term was introduced into the model. Height, weight or BMI was not significant predictors for FEV1/FVC ratios.

Measured and predicted (according to ECSC and GLI equations of this current study) FEV1, FVC values, along with the corresponding FEV1/FVC ratios, are summarized in Table **2a** and **2b** for healthy and COPD individuals, respectively.

In the healthy individual's group, the agreement between measured and predicted FEV1 values, based on either the ECSC or GLI or the locally derived equations, was very good with our predicted values having a slightly better agreement (Table **3a**). Likewise, predicted FEV1 and FVC values, derived either by the ECSC or locally derived equations, showed a high correlation and excellent agreement in the group of COPD patients (Table **3b**).

All COPD patients were subsequently categorized according to GOLD criteria in 4 stages, with the use of our predicted, ECSC and GLI equations for FEV1. These classifications are shown in Table **4**. Based on ECSC values 17 patients (13.7%) were classified as having mild airflow limitation, 48(38.7%) moderate, 40(32.26%) severe and 19 (15.32%) very severe. The results of the same classification based on our derived predicted values are 17(13.7%),44(35.5%), 42(33.9%) and 21(16.9%), respectively, and for GLI values 14(11.3%),39(31.5%),50(40.3%)and 21(16.9%).

There is an overall agreement among the three classifications: classifications were the same for 93.6% (116/124) of COPD patients with the weighted kappa coefficient of agreement being 0.936 (*p*<0.001)for our predicted, and ECSC values, for our predicted and GLI values classifications were the same for 89.5%(110/124)of COPD patients with the weighted Kappa coefficient of agreement being 0.895(*p*<0.001). Moreover for ECSC and GLI values, classifications were the same for 86.5%(107/124) of COPD patients with the weighted Kappa coefficient of agreement being 0.862.(*p*<0.001)

## 4 DISCUSSION

COPD criteria for diagnosis, staging and stratification are constantly under evaluation. Traditionally, the diagnosis of COPD is based on lung function tests and clinical criteria such as smoking, sputum, cough and shortness of breath. For optimal evaluation of COPD, two spirometric parameters are mainly used - FEV1 and FVC obtained after bronchodilatation. The values used in our equations were standardized by using only one device, by the same technique, taken in the same laboratory during the morning. Ethnicity is an important variable of complex identification. It is understood that ethnicity affects body proportions, such as the Cormic index, which is the relation between the height measured at sitting position (encephalic-trunk height) and standing height.

Lung volume would be more correlated with seating height than with standing height (stature).Leading medical societies have long incorporated race or ethnic “correction” or “adjustment” of lung capacity measurement into their clinical practice guidelines, generally for people considered to be “black”. By 1990, the application of a correction factor (generally 6–12%) or the use of population-specific standards, both of which could be programmed into the spirometer, being commonplace in pulmonary training programmes in the USA [18]. However, the most recent guidelines, published in 2005 by the Joint Working Party of the American Thoracic Society (ATS)/European Respiratory Society (ERS) recommend the use of race- and ethnic-specific reference standards, rather than an adjustment factor, using self-identification to determine race [19].

Reference equations derived from spirometry data locally collected by well-trained personnel might be more appropriate for everyday use than generally used equations based on data from scientific studies in the distant past. Likewise, the purpose of this study was to present equations, for the basic functional parameters (FEV1 and FVC) for the Greek population using an alternate method in order to validate the present reference values for spirometry, obtain new numbers and if different, classify COPD patients in different groups(A,B,C,D)or support data from international literature. Greek population has different anthropometrics characteristics than the ones, whose equations were obtained (little bit shorter than the people from Northern Europe(mean height around 175cm) with a big percentage of the population overweighted or obese(7 out of 10 over 18 yrs old).

For the development of equations predicting FEV1 and FVC, various demographic and anthropometric factors known to predict lung function were taken into consideration. These factors included gender, age, height, and weight. They were considered from the beginning as covariates of the respiratory function. Therefore, their effect was examined. The predicted values of FEV1 and FVC derived from the healthy group of subjects seem to be non-significantly different to the ECSC and GLI predicted values. Interestingly, it seems that there is a better agreement between the reference values derived from the predictive equations and the measured values in this healthy local population. It should be noted that the 3 sets of equations present a non-significant difference. This does not cause a significant difference in the classification of COPD patients. Some small variations in the classification of the patients may be due to the normal standard deviation that causes a different staging of airflow limitation. Also the presence of some differences in the lower limit of normal, with the presence of a higher fluctuation of predicted values, may cause the same effect.

In this study, only the spirometric parameters were used for the classification according to GOLD 2013. It is shown that the 3 sets of values are almost identical, despite the fact that they have been developed from different study populations. It seems, according to the study, that there is a discrepancy in the COPD classification of COPD patients between our study results, ECSC and GLI results.

There is one limitation of our study. The study population pool was from the region of Attica only, not from all the regions of Greece. There is another study conducted in 2011 based on sample from Northern Greece. The equations derived from the study by Kontakiotis *et al*. [20] differed significantly from those predicted using previously published reference equations and suggested that new locally derived spirometry reference equations may be more suitable for evaluation of lung function in everyday practice. According to our results, there is no further need for trying to have normal predictive values for each European country separately. Perhaps a general discussion and cooperation among health institutes throughout Greece will be valuable in order to reach conclusions. Another major problem nowadays is that Europe, including Greece, is facing immigration. People from different ethnicities and races arrive in Europe and a crucial question arises *i.e.* how to classify these COPD patients, with local or international equations. It seems that it is better to use international equations.

2017 GOLD Report [21] separates spirometric grades from the “ABCD” groups. Thus, ABCD groups and their associated implications for pharmacotherapy recommendations will be derived exclusively from patient symptoms and their history of exacerbations. The separation of airflow limitation from clinical parameters makes it clearer what is being evaluated and ranked. This revised assessment tool acknowledges the limitations of FEV1 in influencing some therapeutic decisions for individualized patient care and highlights the importance of patient symptoms and exacerbation risks in patients with COPD. Spirometry remains key in the diagnosis, prognostication and treatment with nonpharmacologic therapies. In this report and in this study, spirometry remains a key in diagnosis, prognostication and treatment of COPD but for now is not in the first line.

Recent criteria for the classification of COPD require not only spirometric indices, but also other clinical parameters such as questionnaires about respiratory exacerbations and symptoms. A clinical index quite complex but highly sensitive concerning the severity of the disease, is BODE (Body-mass index, airflow Obstruction, Dyspnea, and Exercise) [22]. A simpler index has also been proposed, named CPI (COPD Prognostic Index), which includes mortality, exacerbations and hospitalization of patients with COPD.It is obvious that prospective longitudinal cohort studies [23, 24] and randomized, controlled trials are needed to validate this risk assessment tool and scoring system to refine the identification of patients at increased exacerbation risk.

## Figures and Tables

**Table 1:** Anthropometric characteristics of a) the healthy study population and b) COPD patients.

a)				
	**All subjects** **(N=500)**	**Men** **(N = 239)**	**Women** **(N = 261)**	***p*-value**
	**Mean (SD)**	**Mean (SD)**	**Mean (SD)**	
**Age (years)**	47.59 (16.70)	46.92 (16.16)	48.20 (17.19)	0.390
**Height (cm)**	168.96 (9.74)	175.80 (7.25)	162.69 (7.18)	<0.001
**Weight (Kg)**	76.97 (17.20)	84.90 (4.90)	69.70 (15.92)	<0.001
BMI*(Kg/m^2^)	26.91 (5.44)	27.44 (4.38)	26.42 (6.22)	0.036
*Body Mass Index				
b)				
	**All subjects** **(N=124)**	**Men** **(N = 84)**	**Women** **(N = 40)**	***p*-value**
	**Mean (SD)**	**Mean (SD)**	**Mean (SD)**	
**Age (years)**	62.82 (13.16)	62.65 (12.82)	63.18 (14.00)	0.835
**Height (cm)**	167.93 (8.97)	172.17 (6.54)	159.03 (6.56)	<0.001
**Weight (Kg)**	74.55 (16.08)	79.20 (15.73)	64.78 (12.00)	<0.001
BMI* (Kg/m^2^)	26.34 (4.74)	26.68 (4.79)	25.62 (4.60)	0.246
Data are presented as mean (standard deviation); *Body Mass Index				

**Table 2:** Measured and predicted (by ECSC,GLI and current study’s equations) FEV1, FVC values along with the corresponding FEV1/FVC (%) ratios for a) the group of healthy individuals and b) COPD patients.

a)				
	**All subjects** **(N=500)**	**Men** **(N = 239)**	**Women** **(N = 261)**	**p-value**
	**Mean (SD)**	**Mean (SD)**	**Mean (SD)**	
Measured values				
*FEV1*	3.20 (0.86)	3.75 (0.73)	2.70 (0.63)	<0.001
*FVC*	3.83 (1.08)	4.55 (0.89)	3.17 (0.76)	<0.001
*FEV1/FVC*	84.26 (8.06)	82.75 (6.90)	85.63 (8.79)	<0.001
Predicted (ECSC equations)				
*FEV1*	3.14 (0.83)	3.70 (0.63)	2.62 (0.62)	<0.001
*FVC*	3.78 (1.00)	4.56 (0.67)	3.06 (0.66)	<0.001
*FEV1/FVC*	79.34 (3.09)	78.72 (2.84)	79.90 (3.21)	<0.001
Predicted (GLI equations)				
*FEV1*	3.33 (0.84)	3.90 (0.67)	2.80 (0.59)	<0.001
*FVC*	4.13 (0.99)	4.88 (0.74)	3.44 (0.64)	<0.001
*FEV1/FVC*	80.85 (2.97)	80.01 (2.77)	81.61 (2.94)	<0.001
Predicted (Study equations)				
*FEV1*	3.20 (0.79)	3.75 (0.62)	2.70 (0.56)	<0.001
*FVC*	3.80 (0.97)	4.52 (0.72)	3.14 (0.65)	<0.001
*FEV1/FVC*	84.26 (2.46)	82.75 (1.14)	85.63 (2.53)	<0.001
**b)**				
	**All subjects** **(N=124)**	**Men** **(N = 84)**	**Women** **(N = 40)**	**p-value**
	**Mean (SD)**	**Mean (SD)**	**Mean (SD)**	
Measured values				
*FEV1*	1.48 (0.74)	1.60 (0.76)	1.22 (0.64)	0.008
*FVC*	2.52 (1.14)	2.79 (1.15)	1.96 (0.90)	<0.001
*FEV1/FVC*	57.90 (10.63)	56.47 (11.39)	60.89 (8.14)	0.030
Predicted (ECSC equations)				
*FEV1*	2.78 (0.71)	3.10 (0.54)	2.10 (0.51)	<0.001
*FVC*	3.48 (0.88)	3.95 (0.58)	2.51 (0.55)	<0.001
*FEV1/FVC*	76.31 (2.47)	75.93 (2.31)	77.09 (2.65)	<0.001
Predicted (GLI equations)				
*FEV1*	2.95 (0.70)	3.26 (0.57)	2.30 (0.48)	<0.001
*FVC*	3.78 (0.86)	4.20 (0.65)	2.91 (0.53)	<0.001
*FEV1/FVC*	78.16 (2.14)	77.59 (1.93)	79.35 (2.08)	<0.001
Predicted (Study equations)				
*FEV1*	2.86 (0.68)	3.17 (0.55)	2.23 (0.46)	<0.001
*FVC*	3.50 (0.83)	3.90 (0.60)	2.65 (0.54)	<0.001
*FEV1/FVC*	82.62 (1.88)	81.64 (0.91)	84.69 (1.72)	<0.001

**Table 3:** Concordance between a) predicted (ECSC, GLI-2012 and local study equations) and measured FEV1, FVC and FEV1/FVC (%) ratios in healthy individuals and b) predicted by ECSC, GLI-2012 and local study equations FEV1, FVC and FEV1/FVC (%) ratios in COPD individuals.

a)									
	**All subjects** **(N=500)**			**Men** **(N=239)**			**Women** **(N=261)**		
	**ECSC**	**GLI**	**Local** **study**	**ECSC**	**GLI**	**Local** **study**	**ECSC**	**GLI**	**Local** **study**
**FEV1**									
*Lin’s coefficient ρ_c_*	0.917	0.910	0.920	0.838	0.829	0.844	0.886	0.874	0.888
Average difference*	-0.067	0.126	0.000	-0.053	0.145	0.000	-0.079	0.108	-0.000
*Pearson's coef. r*	0.921	0.920	0.923	0.849	0.849	0.854	0.893	0.889	0.893
*R^2^*	0.848	0.846	0.852	0.721	0.722	0.730	0.798	0.790	0.798
**FVC**									
*Lin’s coefficient ρ_c_*	0.887	0.855	0.893	0.759	0.722	0.789	0.809	0.762	0.822
Average difference*	-0.055	0.297	-0.027	0.006	0.324	-0.030	-0.110	0.273	-0.025
*Pearson's coef. r*	0.890	0.893	0.898	0.788	0.792	0.807	0.827	0.831	0.833
*R^2^*	0.792	0.798	0.807	0.622	0.628	0.652	0.684	0.691	0.694
**FEV1/FVC**									
*Lin’s coefficient ρ_c_*	0.097	0.139	0.170	0.089	0.102	0.053	0.077	0.123	0.153
Average difference*	-4.919	-3.411	0.000	-4.033	-2.739	-0.000	-5.731	-4.025	0.000
*Pearson's coef. r*	0.193	0.249	0.305	0.164	0.167	0.166	0.165	0.243	0.288
*R^2^*	0.037	0.062	0.093	0.027	0.028	0.027	0.027	0.059	0.083
*Predicted – measured									
**b)**									
	**All subjects** **(N=124)**			**Men** **(N = 84)**			**Women** **(N = 40)**		
	**ECSC** ***vs*.** **Local eqs.**	**GLI** ***vs*.** **Local eqs.**	**ECSC** ***vs*.** **GLI eqs.**	**ECSC** ***vs*.** **Local eqs.**	**GLI** ***vs*.** **Local eqs.**	**ECSC** ***vs*.** **GLI eqs.**	**ECSC** ***vs*.** **Local eqs.**	**GLI** ***vs*.** **Local eqs.**	**ECSC** ***vs*.** **GLI eqs.**
**FEV1**									
*Lin’s coefficient ρ_c_*	0.989	0.987	0.967	0.988	0.976	0.955	0.962	0.983	0.916
Average difference*	-0.089	0.083	-0.172	-0.071	0.088	-0.160	-0.127	0.071	-0.197
*Pearson's coef. r*	0.998	0.995	0.996	0.997	0.990	0.997	>0.999	0.995	0.993
*R^2^*	0.996	0.989	0.992	0.994	0.979	0.993	>0.999	0.990	0.987
**FVC**									
*Lin’s coefficient ρ_c_*	0.988	0.932	0.937	0.979	0.869	0.912	0.965	0.875	0.779
Average difference*	-0.011	0.287	-0.298	0.047	0.299	-0.252	-0.134	0.262	-0.396
*Pearson's coef. r*	0.991	0.987	0.992	0.982	0.972	0.995	0.995	0.983	0.993
*R^2^*	0.981	0.974	0.985	0.965	0.944	0.989	0.990	0.967	0.986
**FEV1/FVC**									
*Lin’s coefficient ρ_c_*	0.093	0.190	0.713	0.107	0.160	0.730	0.012	0.061	0.639
Average difference*	-6.317	-4.466	-1.852	-5.707	-4.050	-1.657	-7.599	-5.340	-2.259
*Pearson's coef. r*	0.499	0.666	0.953	>0.999	0.969	0.969	0.092	0.309	0.960
*R^2^*	0.249	0.443	0.909	>0.999	0.939	0.938	0.008	0.096	0.922
*Differences calculated as ECSC-Local Eqs., GLI-Local Eqs. and ECSC-GLI, respectively									

**Table 4:** Cross tabulation of GOLD staging classifications of the COPD patients based on predicted values from the ECSC, GLI-2012 and the local study equations. Cases of disagreement are shown in bold font.

**GOLD staging** **(ECSC based)**					
	*Mild*	*Moderate*	*Severe*	*Very severe*	Overall
**GOLD staging (local study based)**	**N (%)**	**N (%)**	**N (%)**	**N (%)**	**N (%)**
***Mild***	16 (94.1)	**1 (2.1)**	0 (0.0)	0 (0.0)	17 (13.7)
***Moderate***	**1 (5.9)**	43 (89.6)	0 (0.0)	0 (0.0)	44 (35.5)
***Severe***	0 (0.0)	**4 (8.3)**	38 (95.0)	0 (0.0)	42 (33.9)
***Very severe***	0 (0.0)	0 (0.0)	**2 (5.0)**	19(100.0)	21 (16.9)
**Overall**	17 (13.7)	48 (38.7)	40 (32.3)	19(15.3)	124(100)
93.6% agreement; weighted Kappa=0.936					
**GOLD staging** **(GLI based)**					
	*Mild*	*Moderate*	*Severe*	*Very severe*	**Overall**
**GOLD staging (local study based)**	**N (%)**	**N (%)**	**N (%)**	**N (%)**	**N (%)**
***Mild***	13 (92.9)	**4 (10.3)**	0 (0.0)	0 (0.0)	17 (13.7)
***Moderate***	**1 (7.1)**	35 (89.7)	**8 (16.0)**	0 (0.0)	44 (35.5)
***Severe***	0 (0.0)	0 (0.0)	42 (84.0)	0 (0.0)	42 (33.9)
***Very severe***	0 (0.0)	0 (0.0)	0 (0.0)	21 (100.0)	21 (16.9)
**Overall**	14 (11.3)	39 (31.5)	50 (40.3)	21 (16.9)	124 (100.0)
89.5% agreement; weighted Kappa=0.895					
**GOLD staging** **(GLI based)**					
**-**	*Mild*	*Moderate*	*Severe*	*Very severe*	**Overall**
**GOLD staging (ECSC based)**	**N (%)**	**N (%)**	**N (%)**	**N (%)**	**N (%)**
***Mild***	14 (100.0)	**3 (7.7)**	0 (0.0)	0 (0.0)	17 (13.7)
***Moderate***	0 (0.0)	36 (92.3)	**12 (24.0)**	0 (0.0)	48 (38.7)
***Severe***	0 (0.0)	0 (0.0)	38 (76.0)	**2 (9.5)**	40 (32.3)
***Very severe***	0 (0.0)	0 (0.0)	0 (0.0)	19 (90.5)	19 (15.3)
**Overall**	14 (11.3)	39 (31.5)	50 (40.3)	21 (16.9)	124 (100.0)
86.3% agreement; weighted Kappa=0.862					
